# Annotations

**Published:** 1888-09-15

**Authors:** 


### Annotations.

**The Rich and the Whitechapel Murders.**

The murders in Whitechapel and their accompanying circumstances of horror have called the attention of the metropolis and of the country to the character and mode of life of a large portion of the population of London. There is no necessity to exaggerate facts or to heighten effects by untruthful colouring. We see, beyond possibility of question, that tens of thousands of our people are in a state of mental insensibility and moral degradation which it is as difficult to realise as it is easy to believe. At the East End, and in other working-class districts, the mere struggle for bread and lodging is more than sufficient to exhaust the mental and physical energies of the people. When the bread, and the covering for the body, and the bed for the night have been secured, there is no strength or disposition left either for thought or hope. All the variety that is possible is some form of exciting amusement. Some of us who have leisure and opportunities must lay these facts to heart. It is impossible to throw the responsibility upon policemen or statesmen or any other official class. The present result has been brought about by the prevalent economic conditions, and by the almost complete indifference of the more prosperous to the distresses and straits of their hard-driven fellow citizens. To say that the indifference of the better classes is unbrotherly and un-Christian is to use words that have no fitness for the occasion. It is inhuman, it is shameful, and it is suicidal. If the toes of a man, which are situated at the extremest limits of his organism, and which one would think are among the least essential parts of the body, take to mortifying, what do we expect? The speedy death of the man! And does any sane person consider that the lower strata of society can become corrupt, degraded, and morally and physically worthless, without producing a corresponding effect on the whole social organism? It is an elementary fact in political economy that society as a whole consists of all its parts. No one part can be well or ill, strong or weak, base or noble alone. Those who are wealthy but idle, intelligent but cynical, sympathetic but helpless, deserve the severest reprobation which language can express. Fools and suicides it is evident that they are. Can any one doubt that there is wealth enough in London, intelligence enough, and even good feeling enough to turn the worst pandemonium of the East End into a prosperous and self-respecting region? Here is the power and there is the work. "The power wants direction," is it said? But the power is blessed with intelligence—with ten thousand separate intelligences. If there is no central directing authority which can set it to work as a whole, why does not each intelligence set itself to work? The plain truth is that the upper strata of society are to a large extent as self-indulgent, self-centred, and morally worthless as the lower; and without one-hundredth part of the justification which may be pleaded for the poor. If Christianity is a failure among the poor, it is still more a failure among the rich. Where one man recognizes and strives to do his duty by his fellows, five hundred others use the world for no other purpose but for the personal success and pleasure they can get out of it. As moral beings and parts of the divinely-appointed social order they are beneath contempt. It is a safe prophecy that this kind of madness and inhumanity cannot continue for ever.

**Murdered Children.**

Students of the daily newspapers sometimes wonder what particular advantage or pleasure it can bring to a higher Power to preserve the human race alive. The stories of children, helpless and friendless, murdered in all kinds of ways by their parents and guardians for the sake of an insignificant sum which even the poorest might earn by honest labour in a few weeks —these stories reveal a condition of mind in most beings so desperate and so hopeless that some moods would willingly consign to everlasting oblivion the whole race which has given origin to such more than fiendish fiends. Even the devil, one would think, would by this time have become surfeited with the sight of such monstrous and incredible deeds, and would cease to tempt the wretches who are capable of them. There are certain people who are always objecting to severe punishments, even for proved crime. What punishments can be severe enough for those who deliberately and of set purpose insure the lives of their own children in order that they may immediately set about destroying them? We confess to a feeling of intense savageness when we read the stories of such unnatural crimes, which makes us regard with satisfaction the possibility even of eternal punishment in the world to come. The mere hanging of a man who slowly kills his helpless infant by starvation, poison, or cruel bodily injury is as nothing compared with his deserts. The punishment should be so severe and so long continued as really to open his eyes to the enormity of his own deeds. The moral sense of the community at the present time does not seem to be wanting in keenness; the public conscience does not appear to us to be less active, but more active, than it was in the days of Dante. Still, the protection afforded to infant life is not merely deficient, but in many cases appears to be *nil*. The natural protectors of child-life are, of course, the parents, and among the cattle and the wild animals no other protectors are needed. But a portion of human society has fallen to a level of degradation and cruelty which, by comparison with the condition of wild animals, is immeasurably infamous. The deeds of baby-farmers which come to light, and the still more cruel and incredible barbarities which never see the light, are enough to make the most hopeful despair. We owe it to ourselves as a people to take steps, if not entirely to prevent, at least to reduce to a minimum, these unendurable crimes. Inquiry must be made into the doings of Insurance Offices. Measures must be taken for the control and regulation of their business. Monsters who are discovered in the perpetration of these blackest of all crimes must be punished, not, indeed, as they deserve, for that is not possible, but with the sternest penalties known to human law; but above all, those in whose hearts glow the passion of a divine love should make some effort to deal with the terrible tendencies of the times. The good and the cultured among us ought by all means in their power to strive to resist this downward movement among the brutal and the ignorant. Punishment alone has always been futile to reform. The mind and character must be instructed and improved. For this there is only one way. The personal contact, and persuasion, and influence of those who are intelligent and virtuous can alone bring about a change. It is for each one of us to take his share of the task.

**Doctors and Chemists.**

A discussion is now going on in the *Daily Chronicle* on the respective provinces of doctor and chemist, or rather, on their invasions of each other's province. One set of correspondents condemn the prescribing chemist, while the others say hard things of the dispensing doctor. Some of the chemists protest that they have often to use their knowledge and accumulated experience in altering the prescription of a doctor, who otherwise would, quite unwittingly, kill his patient. The doctors, on the other hand, declare that the hardest task they have ever set them is the cure of some hapless mortal whom the prescribing chemist has treated wrongly. Doubtless there is some measure of truth in both accusations; it is equally certain that too much is being made of them. It is foolish on the part of the doctors to depreciate too much the chemist's knowledge, or to place him on the same level as the grocer or the butterman. He has passed two examinations, the first of which proves him to be possessed of a good education, while the second has tested his knowledge of therapeutics. If a chemist knows the disease from which any patient is suffering, it is highly probable, if he is an intelligent man, that he will have some notion of the proper treatment of it. But in diagnosis he is apt to fail, for his training has not taught him to differentiate between one set of symptoms and another, which, to the inexperienced eye, seem nearly identical. Yet it is natural that he should wish to use the knowledge he does possess, and should resent being confined, like a hairdresser, to the sale of soaps, scents, and hair-oil. He asks, therefore, that the doctor should confine himself to prescribing, and leave the dispensing to chemists. On the other hand, the doctor says that his patients feel swindled if they get only advice for their money, without the addition of something real and tangible—the bottle of medicine. As a matter of fact, both are too much tempted to give medicine where none is needed, and we must look to the formation of a wiser public opinion to give a proper and deserved place to each.

